# Zdhhc2 Is Essential for Plasmacytoid Dendritic Cells Mediated Inflammatory Response in Psoriasis

**DOI:** 10.3389/fimmu.2020.607442

**Published:** 2021-01-08

**Authors:** Binhui Zhou, Wenyi Yang, Wushan Li, Le He, Liaoxun Lu, Lichen Zhang, Zhuangzhuang Liu, Ying Wang, Tianzhu Chao, Rong Huang, Yanrong Gu, Tingting Jia, Qiaoli Liu, Shuanghua Tian, Philippe Pierre, Takahiro Maeda, Yinming Liang, Eryan Kong

**Affiliations:** ^1^ Laboratory of Mouse Genetics, Institute of Psychiatry and Neuroscience, Xinxiang Medical University, Henan, China; ^2^ Laboratory of Genetic Regulators in the Immune System, Henan Collaborative Innovation Center of Molecular Diagnosis and Laboratory Medicine, Xinxiang Medical University, Henan, China; ^3^ Henan Key Laboratory of Immunology and Targeted Therapy, School of Laboratory Medicine, Xinxiang Medical University, Henan, China; ^4^ Centre d’Immunologie de Marseille-Luminy (CIML), INSERM, CNRS, Aix Marseille Université, Marseille, France; ^5^ Department of Medical Sciences, Institute for Research in Biomedicine (iBiMED) and Ilidio Pinho Foundation, University of Aveiro, Aveiro, Portugal; ^6^ Department of Microbiology and Immunology, Shanghai Institute of Immunology, Shanghai Jiao Tong University School of Medicine, Shanghai, China; ^7^ Department of Island and Community Medicine, Island Medical Research Institute, Nagasaki University Graduate School of Biomedical Sciences, Nagasaki, Japan

**Keywords:** psoriasis, autoimmunity, Zdhhc2, plasmacytoid dendritic cells, inflammatory response

## Abstract

Zdhhc family genes are composed of 24 members that regulate palmitoylation, a post-translational modification process for proteins. Mutations in genes that alter palmitoylation or de-palmitoylation could result in neurodegenerative diseases and inflammatory disorders. In this study, we found that Zdhhc2 was robustly induced in psoriatic skin and loss of Zdhhc2 in mice by CRISPR/Cas9 dramatically inhibited pathology of the ear skin following imiquimod treatment. As psoriasis is an inflammatory disorder, we analyzed tissue infiltrating immune cells and cytokine production. Strikingly we found that a master psoriatic cytokine interferon-α (IFN-α) in the lesioned skin of wildtype (WT) mice was 23-fold higher than that in Zdhhc2 deficient counterparts. In addition, we found that CD45^+^ white blood cells (WBC) infiltrating in the skin of Zdhhc2 deficient mice were also significantly reduced. Amelioration in psoriasis and dramatically reduced inflammation of Zdhhc2 deficient mice led us to analyze the cellular components that were affected by loss of Zdhhc2. We found that imiquimod induced plasmacytoid dendritic cell (pDC) accumulation in psoriatic skin, spleen, and draining lymph nodes (DLN) were drastically decreased in Zdhhc2 deficient mice, and the expression of pDC activation marker CD80 also exhibited significantly inhibited in psoriatic skin. In further experiments, we confirmed the cell intrinsic effect of Zdhhc2 on pDCs as we found that loss of zDHHC2 in human CAL-1 pDC dampened both interferon regulatory factor 7 (IRF7) phosphorylation and IFN-α production. Therefore, we identified novel function of Zdhhc2 in controlling inflammatory response in psoriasis in mice and we also confirmed that crucial role of Zdhhc2 in pDCs by regulating IRF7 activity and production of the critical cytokine. Our results finding the dependence of IFN-α production on Zdhhc2 in inflamed murine skin and in human pDCs provide rationale for targeting this new molecule in treatment of inflammation.

## Introduction

Psoriasis is the most common chronic autoimmune skin disease in humans, affecting about 2~3% of the worldwide population, and seriously affects the physical and mental health of patients (1). The severity of the disease depends on inheritance and environmental factors, mild patients have isolated scaly erythematous plaques on the scalp, knees, or elbows, whereas severe patients have up to 100% of the cutaneous surface (2). Some studies demonstrated that psoriasis is a disease mediated by T cells and dendritic cells, and IL-23 and IL-12 were released by inflammatory myeloid dendritic cells to activate helper T cells to produce abundant pro-inflammatory cytokines as IFN-γ, IL-17, and IL-22 (3, 4). And other research have shown that pDC plays an important role in initiating psoriasis through interferon-alpha production (5).

Published studies have shown that zDHHC family genes are closely related to the occurrence of various diseases including inflammatory disorders (6–9), and psoriasis is one of the inflammatory skin diseases mediated by the immune cells and molecules of the innate and adaptive immune systems (10). On this basis, we performed screening for zDHHC family genes for their involvement in psoriasis (unpublished data), because such family of genes regulate a highly conserved biochemical process termed palmitoylation which exist in yeasts as well as in mammals (9, 11, 12). More importantly little is known whether zDHHC family genes are essential for pathogenesis of psoriasis.

In this study, we found that the mRNA level of *Zdhhc2* was significantly elevated in mice inflamed skin upon imiquimod-induced psoriasis. Knockout of *Zdhhc2* in mice potently inhibited pathological grade and infiltration of inflammatory cells in the skin. In addition, we observed that Zdhhc2 deficiency dramatically reduced the expression level of pro-inflammatory cytokine IFN-α in the inflamed skin, and the accumulation and activation state of pDCs were significantly inhibited in imiquimod challenged skin of Zdhhc2 deficient mice. Moreover, loss of zDHHC2 in human pDC cell line termed CAL-1 dampened IRF7 activity. The functional identification of Zdhhc2 and mechanistic discoveries in our study provide an insight into possibilities of manipulating the enzymatic activity of zDHHC2 for treatment of skin inflammatory diseases such as psoriasis.

## Materials and Methods

### Mice

C57BL/6 mice were purchased from Beijing Vital River Laboratory Animal Technology Co., Ltd. CD45.1^+^ CD3ϵ^−/−^ mice without T cells were generated as described previously (13). All animals were kept in SPF environment with a 12-h light, 12-h dark cycle and free access of animals to food and water.

### Reagents

The TLR7 agonist gardiquimod (Sigma) was dissolved in DMSO (Sigma) at a concentration of 100 μg/ml and stored in aliquots at −20°C. Gardiquimod was added to cell cultures at a working concentration of 0.5 μg/ml. Antibodies for western blot include primary antibodies: rabbit anti-phospho-p65 (Ser536) (3033, Cell Signaling Technology), rabbit anti-p65 (4764, Cell Signaling Technology), rabbit anti-TLR7 (ab24184, Abcam), mouse anti-Lamp1 (H4A3, Santa Cruz Biotechnology), rabbit anti-IFN-α (YT5170, Immunoway), rabbit anti-GAPDH (AF1186, Beyotime) and secondary antibodies: HRP conjugated goat anti-rabbit IgG (H+L) (A0208, Beyotime), Alexa Fluor 594 goat anti-rabbit IgG (H+L) (A-11012, Thermo Fisher Scientific) and Alexa Fluor 488 goat anti-mouse IgG (H+L) (A-11017, Thermo Fisher Scientific). Antibodies for flow cytometry analysis were listed in **Supplementary Table 1**.

### Generation of Zdhhc2 Knockout Mice Using CRISPR/Cas9 System

For generation of Zdhhc2^−/−^ mice on the background of C57BL/6, two small guide RNAs (sgRNAs) (CAATGTTTGTCTGGTCATACTGG, TTGACACCCTAATGAAACGGAGG) were designed and synthesized according to our previous studies (14, 15). *In vitro* fertilization was performed as described previously (16, 17). The tail tips of F0 mice were used for genomic DNA extraction, then PCR was performed to isolate knockout allele by using primer pairs (F: AAATGAGGGGGTTTATTTATGGA, R: GGACCTAGTGAAATTTCCATCTTT) which were flanking beyond the two sgRNA targeted sites. PCR products were further subcloned into pBLUE-T (Beijing Zoman Biotechnology Co., Ltd, ZC204) and analyzed by sanger sequencing.

### Generation of Imiquimod-Induced Psoriasis Mouse Model

Eight to eleven weeks old male mice (C57BL/6 and Zdhhc2^−/−^) were divided into three groups, the control group and imiquimod groups subjected to continuous 4 or 8 days of treatment. Imiquimod cream (14 mg, 5% imiquimod) was applied daily on both sides of the ear skin of the mice in the treated groups. No treatment was performed for the control mice. The Psoriasis Area Severity Index was used to monitor and grade the severity of ear skin inﬂammation in mice. Erythema and scaling were scored independently on a scale from 0 to 4 (0, none; 1, slight; 2, moderate; 3, marked; and 4, very marked).

### Histology and Immunohistology

Six mice from each group were sacriﬁced after imiquimod treatment for 0, 4, and 8 days, respectively. Samples from psoriatic ear skin of mice were paraformaldehyde-fixed and embedded in paraffin, sections of 3 μm were prepared for hematoxylin and eosin (H&E) staining. The inflammatory infiltrate was characterized by immunohistochemistry. Polyclonal antibody specific for CD45 (GB11522, Servicebio) was used as primary antibody, secondary labeling was performed using DAB chromogenic Kit (DAKO). Images were acquired by light microscopy (Nikon DS-U3).

### CRISPR/Cas9 Mediated Knockout of zDHHC2 in CAL-1 Cells

CAL-1 cell line was cultured in 1640 medium supplemented with 10% fetal bovine serum (FBS, HyClone), 100 units/ml penicillin, 100 µg/ml streptomycin, 1 mM sodium pyruvate (Gibco), 10 mM HEPES (Gibco), 1% non-essential amino acid (100×, Millipore), 1% glutamine (100×, Gibco) and 0.1% β-mercaptoethanol (Sigma), incubated in % CO_2_ at 37°C. To generate zDHHC2^−/−^ CAL-1 cell lines using CRISPR/Cas9 system, two sgRNAs (TCGCCTAAGAACTTCCTGATGGG, TATACCAGGACCATGTCTGGAGG) were designed and synthesized for human *zDHHC2* gene and respectively cloned into pX458 vector with EGFP which enabled single cell sorting as described previously (18). CAL-1 cells were co-electroporated with plasmids of px458-zDHHC2-sgRNA1 and px458-zDHHC2-sgRNA2 by using a Neon^®^ Transfection System (Thermo Fisher Scientific). Two days after electroporation, single cell was sorted by BD FACSAria™ Fusion and cultured until colonies were formed. Individual colonies were picked and screened by PCR using primer pairs (F: 5’-TCCGGGTATGGTAGAGAAGAC-3’, R: 5’-ACACACTTTCTTACAGTCACCT-3’), and sanger sequencing was performed for further verification.

### Quantitative Real-Time Polymerase Chain Reaction (qRT-PCR)

Total RNA was extracted from partial lesioned skin of ear from WT and zDHHC2^−/−^ mice or CAL-1 WT and CAL-1 zDHHC2^−/−^ cells using the RNeasy Plus Mini Kit (Qiagen). cDNA was prepared using MEGAshortscript™ T7 Transcription Kit (Thermo Fisher Scientific). The mRNA levels of Zdhhc2, IFN-α, tumor necrosis factor-α (TNF-α), interleukin-23 (IL-23), interleukin-17a (IL-17a), and hypoxanthine-guanine phosphoribosyltransferase (HPRT) in mice and IFN-α, GAPDH in CAL-1 cells were detected by qRT-PCR analysis based on TB Green Premix Ex TaqTM (TaKaRa) using the Applied Biosystems ABI 7500. The sequences for the qRT-PCR primers were listed in **Supplementary Table 2**. The expression levels were normalized for HPRT mRNA level in mice and GAPDH mRNA level in CAL-1 cells, and the value of 2^-ΔCt^ was used to determine the fold changes between samples.

### Cell Isolation, Adoptive Transfer, and Imiquimod Treatment

Lymph nodes from CD45.2^+^ C57BL/6 and CD45.2^+^ Zdhhc2^−/−^ mice were respectively grounded and filtered through a 70 μm cell strainer into PBS to obtain cell suspensions. The negative selection immunomagnetic cell separation method was used to purify T cells by incubating with antibody mixture including CD11b-biotin (13-0112-85, eBioscience), CD19-biotin (13-0193-85, eBioscience), CD11c-biotin (13-0114-85, eBioscience), and Dynabeads™ M-280 Streptavidin (11206D, Invitrogen). Then the purified CD45.2^+^ C57BL/6 or CD45.2^+^ Zdhhc2^−/−^ T cells were counted, and 2 million cells were transferred intravenously into the CD45.1^+^ CD3ϵ^−/−^ mice, respectively. After 7 days, the transferred mice were treated with imiquimod for continuous 7 days. Then the lesioned skin were collected for flow cytometry analysis.

### Flow Cytometry

Four types of tissues including blood, DLN, spleen, and ear skin were collected from steady state and 8 days imiquimod-treatment mice. Blood was treated with 1× RBC solution (00-4333-57, eBioscience) to lyse red blood cells. Spleen and DLN were dissociated in RPMI 1640 medium containing 10% FBS, 200 μg/ml Collagenase IV (DN25, Sigma), and 100 μg/ml DNase I (C5138, Sigma) with the gentleMACS™ Octo Dissociator, and then incubated at 37°C, 200 rpm for 15 and 30 min, respectively. Mouse psoriatic skin was cut into small pieces of 2~4 mm with scissors and incubated for 100 min at 37°C, 180 rpm with basic Hank’s Balanced Salt Solution (HBSS, 14065-056, Gibco) containing 250 μg/ml Liberase TL (5401020001, Roche) and 500 μg/ml DNase I, then transferred into the gentleMACS C tube to dissociation with the gentleMACS™ Octo Dissociator at 37°C for 5 min. All samples were respectively filtered into 15 ml tubes with MACS SmartStrainer (30 μm) to obtain single cell suspension. The cells were collected by centrifugation and resuspended, 10 μl cells were used for CD45-FITC (MABF320, Millipore) staining and counting. Then 1 million CD45^+^ cells of blood samples, 3 million CD45^+^ cells of spleen and DLN samples, and all the cells of skin samples were respectively stained with antibody mixture, 20 μl absolute counting beads were added in each skin samples. Afterward, 0.5~1 million cells of blood, spleen and DLN samples while 1~2 million cells of skin samples were analyzed using an FACS Canto II or FACSymphony A5 system using FlowJo 10.0 software (BD Biosciences).

To determine the expression of TLR7 in CAL-1 WT and CAL-1 zDHHC2^−/−^ cells, 1 million cells per well were cultured in 12-well plate with gardiquimod stimulation for 0, 12, and 24 h. After stimulation, the cells were fixed with IC fixation buffer (eBioscience) and permeabilized with 1× permeabilization buffer (eBioscience) at 4°C for 30 min. Then the cells were incubated with primary antibody for 30 min at 4°C. In order to detect the phosphorylation level of IRF7 and p65, CAL-1 WT and CAL-1 zDHHC2^−/−^ cells were stimulated with gardiquimod for 0, 60, and 90 min, respectively. Then the cells were fixed with Phosflow lyse/fix buffer (BD Pharmingen) in 37°C water bath for 10 min and permeabilized with methanol on ice for 30 min. Then the cells were respectively incubated with primary antibody and secondary antibody for 30 min at 4°C. Afterward, cells were analyzed using an FACS Canto II system using FlowJo 10.0 software (BD Biosciences).

### Western Blot

CAL-1 WT and CAL-1 zDHHC2^−/−^ cells were cultured in 6-well plate and stimulated with gardiquimod for 0, 60, and 90 min for phosph-IRF7 analysis, or stimulated with gardiquimod for 0 and 24 h meanwhile treated with Golgistop (554724, BD) for 10 h for IFN-α determination. Then the cells were collected and lysed with RIPA lysis buffer for protein extraction. Protein samples were used for western blot analysis as previously described (16).

### Immunofluorescence

After CAL-1 WT and CAL-1 zDHHC2^−/−^ cells were respectively stimulated with gardiquimod for 0 and 24 h, cells were collected in 1.5 ml tubes and fixed with 4% paraformaldehyde for 15 min, permeabilized with 0.1% Triton X-100 in PBS for 10 min, and blocked with 3% BSA in PBST for 1 h at room temperature. Cells were then incubated with rabbit anti-TLR7 (1:500) and mouse anti-Lamp1 (1:100) primary antibodies for 2 h at room temperature. After three times of wash with PBS, cells were incubated with Alexa Fluor 594 goat anti-rabbit IgG secondary antibody and Alexa Fluor 488 goat anti-mouse IgG secondary antibody (1:1,000) for 1 h at room temperature. After washing three times with PBS, cells were smeared evenly on a glass slide and dried, and then mounted with anti-quenching agent. Images were taken using a Leica TCS SP8 STED confocal microscopy.

### Statistics Analysis

The unpaired Student *t* test was used for statistical analysis with GraphPad Prism software (version 6.0) (^*^ *p* < 0.05, ^**^ *p* < 0.01, ^***^ *p* < 0.001, ^****^ *p* < 0.0001).

## Results

### Zdhhc2 Deficiency in Mice by CRISPR/Cas9 Targeting Mitigates Psoriasis

**Figure 1:**
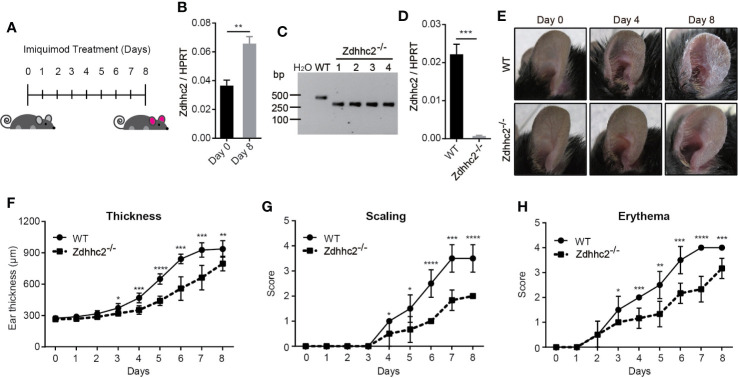
Assessment of psoriasis symptoms induced by imiquimod in *Zdhhc2* knockout mice. **(A)** Schematic representation of imiquimod stimulation on mice ear. **(B)** Zdhhc2 expression level was analyzed by qRT-PCR in ear skin of WT mice with (8 days) or without (0 days) imiquimod treatment (n = 3, mean ± SEM). **(C)** Zdhhc2 knockout mice were screened by PCR using tail genomic DNA of Zdhhc2^−/−^ mice. Results from Zdhhc2^−/−^ mice were shown and compared to WT mice. H_2_O was used as negative control. **(D)** Zdhhc2^−/−^ mice were verified by qRT-PCR analysis (n = 3, mean ± SEM). **(E)** Representative pictures of WT and Zdhhc2^−/−^ mice ears respectively treated with imiquimod for 0, 4, and 8 days. Experiments were repeated three times involving three mice for each treatment group. **(F–H)** Imiquimod was applied on ear skin of WT and Zdhhc2^−/−^ mice for 8 consecutive days, and ear thickness, scaling, and erythema were measured on a daily basis. Scaling and erythema were scored independently from 0 to 4 (see *Materials and Methods*). Two representative experiments are depicted (n = 6, mean ± SEM). ^*^ *p* < 0.05, ^**^ *p* < 0.01, ^***^ *p* < 0.001, ^****^ *p* < 0.0001, unpaired Student’s *t-*test.

Previous studies have shown that zDHHC family genes play important roles in many diseases, such as neurodegenerative diseases, cancer, and inflammation (6, 7, 12). However, characterization of Zdhhc family genes for their contribution to the skin disorders such as psoriasis is still lacking. In the present study, we found that the expression level of Zdhhc2 in mice was potently induced during psoriatic pathogenesis (**Figures 1A, B**). To further gain insight into the function of Zdhhc2 in psoriasis, we used CRISPR/Cas9 system by designing two sgRNAs to target Zdhhc2 in mice (**Supplementary Figure 1A**). PCR amplicons of the mutant mice as well as sanger sequencing and mRNA quantification results showed that we successfully obtained genetic model of Zdhhc2^−/−^ mouse on the pure C57BL/6 background (**Figures 1C, D**, and **Supplementary Figure 1B**). To functionally decipher the role of Zdhhc2 in psoriasis, we applied imiquimod on the ear skin of WT controls and Zdhhc2^−/−^ mice to induce inflammation and compare the pathological changes. Interestingly, we found the typical and severe pathological changes in WT controls mice were obviously ameliorated in the ear skin of Zdhhc2^−/−^ mice after imiquimod treatment for 4 and 8 days (**Figure 1E**). In addition, we quantitated the pathology grade on a daily basis and found that the thickness, scaling, and erythema were significantly decreased in Zdhhc2^−/−^ mice when they were compared to WT controls (**Figures 1F–H**). These results reveled that Zdhhc2 plays an important role in the occurrence of psoriasis.

### Zdhhc2 Deficiency Dramatically Reduces Inflammatory Response During Psoriasis Modeling

**Figure 2:**
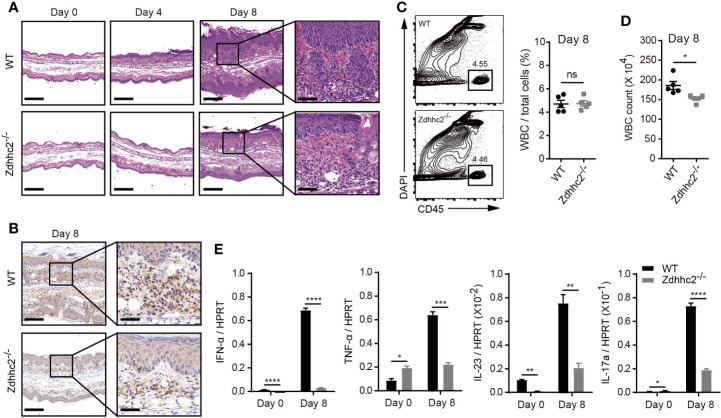
Imiquimod-induced inflammation in Zdhhc2-deficient mice. **(A)** Representative H&E staining on histological sections of WT and Zdhhc2 deficient ears treated with imiquimod for 0, 4, and 8 days, respectively. **(B)** Representative IHC staining for CD45 on histological sections of WT and Zdhhc2 deficient ears treated with imiquimod for 8 days. Image captured at 50× magnification, scale bars: 200 µm. Zoomed-in views of selected regions were captured at 200× magnification, scale bars: 50 μm. Experiments were repeated twice, involving three mice for each time point per genotype. **(C)** The frequency of WBC was analyzed in psoriatic skin of WT and Zdhhc2 deficient mice after 8 days imiquimod treatment. The CD45^+^ single cells were gated with dead cell exclusion using DAPI a nucleic acid staining dye. **(D)** Absolute number of WBC were quantified by Attune NxT volumetric flow cytometer. Experiments were involving five mice for each time point per genotype (mean ± SEM). **(E)** qRT-PCR was performed to analyze the expression level of four pro-inflammation cytokines in WT and Zdhhc2 deficient ears with (8 days) or without (0 days) imiquimod treatment (n = 3, mean ± SEM). ^*^ *p* < 0.05, ^**^ *p* < 0.01, ^***^ *p* < 0.001, ^****^ *p* < 0.0001, unpaired Student’s *t-*test. ns, not significant.

It has been shown that psoriasis is an inflammatory skin disease, and its pathogenesis is closely related to inflammatory cell infiltration and inflammatory cytokines secretion. Among the pro-inflammatory cytokines, IL-23 and IL-12 are mainly produced by dendritic cells, and effector T cells are sources for IFN-γ, TNF-α, and IL-17 production, and type-I interferon is secreted by pDCs (3, 5, 19). To determine the involvement of cytokines and inflammatory response in the mitigated pathology in Zdhhc2 deficient mice, we first compared the severity of inflammation in the ear skin of Zdhhc2^−/−^ mice and WT control mice in steady state and after imiquimod treatment for 4 and 8 days. H&E staining results showed that acanthosis and hyperkeratosis were notably decreased in the mutant mice, and such mitigation in inflammation was more striking after 8 days of imiquimod treatment (**Figure 2A**). The immunohistochemistry results of CD45 showed that accumulation of immune cells was significantly inhibited in the mutant mice (**Figure 2B**). Moreover, we also performed quantitative analyses by flow cytometry, and found that the frequency of WBC exhibited no change whereas the absolute number of WBC had significant decrease in Zdhhc2^−/−^ mice when they were compared with WT controls (**Figures 2C, D**). On the other hand, the mRNA expression level of pro-inflammatory cytokines including IFN-α, TNF-α, IL-23, and IL-17a were dramatically decreased in Zdhhc2 deficient ear skin in comparison to WT counterparts, following 8 days of imiquimod treatment. Strikingly, the expression of IFN-α in lesioned ear skin of Zdhhc2^−/−^ mice was reduced by as much as 23-fold (**Figure 2E**). Our results showed that in mice Zdhhc2 deficiency was able to inhibit accumulation of immune cells in the lesioned skin during psoriasis development and very importantly the production of crucial pro-inflammatory cytokines were also dramatically decreased.

### Plasmacytoid Dendritic Cell Accumulation in Inflamed Skin Is Severely Inhibited in Zdhhc2 Deficient Mice

**Figure 3:**
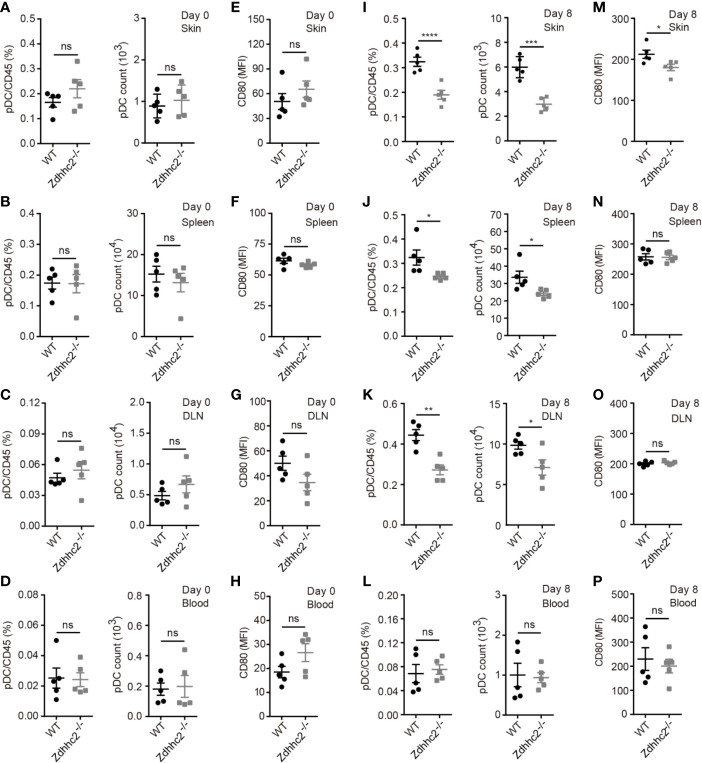
The pDC analysis in four types of organs of WT and Zdhhc2^−/−^ mice following imiquimod treatment for 0 and 8 days. **(A, I)** Comparison of the pDC frequencies in WBC and pDC absolute number in the lesioned skin of WT and Zdhhc2^−/−^ mice after 0 and 8 days imiquimod stimulation. **(E, M)** Mean fluorescence intensity (MFI) of CD80 for pDC in the lesioned skin of WT and Zdhhc2^−/−^ mice after 0 and 8 days imiquimod stimulation. **(B, J)** Comparison of the pDC frequencies in WBC and pDC absolute number in the spleen of WT and Zdhhc2^−/−^ mice after 0 and 8 days imiquimod stimulation. **(F, N)** MFI of CD80 for pDC in the spleen of WT and Zdhhc2^−/−^ mice after 0 and 8 days imiquimod stimulation. **(C, K)** Comparison of the pDC frequencies in WBC and pDC absolute number in the DLN of WT and Zdhhc2^−/−^ mice after 0 and 8 days imiquimod stimulation. **(G, O)** MFI of CD80 for pDC in the DLN of WT and Zdhhc2^−/−^ mice after 0 and 8 days imiquimod stimulation. **(D, L)** Comparison of the pDC frequencies in WBC and pDC absolute number in the blood of WT and Zdhhc2^−/−^ mice after 0 and 8 days imiquimod stimulation. **(H, P)** MFI of CD80 for pDC in the blood of WT and Zdhhc2^−/−^ mice after 0 and 8 days imiquimod stimulation (mean ± SEM). Experiments were involving five mice for each time point per genotype (mean ± SEM). ^*^ *p* < 0.05, ^**^ *p* < 0.01, ^***^ *p* < 0.001, ^****^ *p* < 0.0001, unpaired Student’s *t-*test. ns, not significant.

As we found marked decrease of IFN-α in psoriatic skin, the level of which was regarded as hallmark for initiation of pathology, we postulated that pDCs the major source of IFN-α production could be affected by Zdhhc2 deficiency. It is also important to note that pDCs were found to be involved in psoriasis development in both human and animal models (5, 20–22). However, knowledge of genetic factors governing pDCs and IFN-α production in psoriasis is still quite limited. Hence it is of great interest to determine the contribution of Zdhhc2 to pDCs mediated inflammation in different organs during psoriasis modeling. It has been shown that pDCs circulate in the body through the bloodstream after development in the bone marrow, pDCs are found in the thymus, in secondary lymphoid tissues, and in rare numbers in peripheral tissues under steady state conditions, while pDCs migrate and accumulate in the inflamed tissues after challenge *via* inflammation (23). Thus, we analyzed the presence of pDCs in the skin, spleen, DLN, and blood of WT and Zdhhc2^−/−^ mice following imiquimod treatment for 0 and 8 days, respectively. Under steady state conditions, Zdhhc2 knockout did not affect the frequencies, absolute number, and activation level of pDC in four types of organs (**Figures 3A–H**). After 8 days of imiquimod treatment, the absolute number of pDC was markedly increased in four types of organs compared to that in steady state mice. However, it is interesting to note that the frequencies and absolute number of pDC were significantly lower in lesioned skin, spleen, and DLN of Zdhhc2 deficient mice than in the WT controls, and no change was observed in the blood (**Figures 3I–L**). In addition, compared with WT mice, we observed that the expression of pDC activation marker CD80 was significantly decreased only in the lesioned skin of Zdhhc2^−/−^ mice whereas no such changes were found in the spleen, DLN, and blood (**Figures 3M–P**). Our experiments showed that pDCs an important cellular subset of inflammatory response in psoriasis require the presence of Zdhhc2 to infiltrate and to function in the skin, and the absence of Zdhhc2 notably blocked both gathering of pDCs and development of skin pathology.

### Inhibition of pDC Infiltration by Zdhhc2 Deficiency Impairs T Cell Activation in Psoriatic Skin

**Figure 4:**
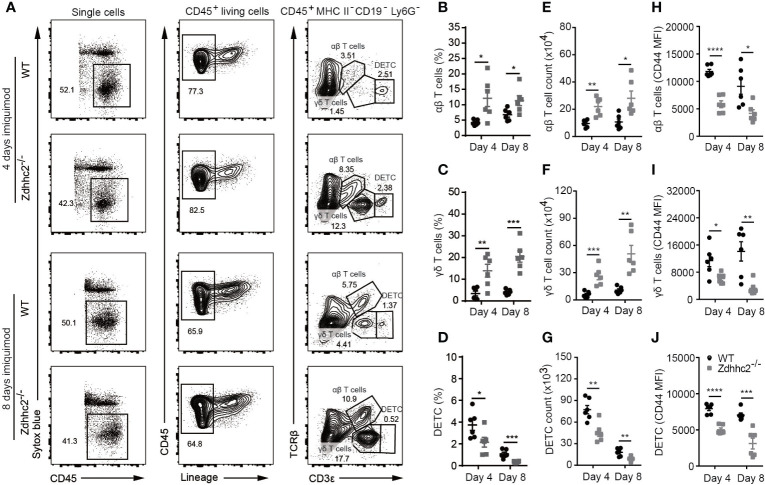
T cell activation in the psoriatic skin of Zdhhc2 deficient mice. **(A)** Three groups of T cell gating strategy in psoriatic skin of WT and Zdhhc2^−/−^ mice after imiquimod treatment for 4 and 8 days, respectively. After excluding dead cells (Sytox blue positive), B cells (CD19^+^), neutrophils (Ly6G^+^), and macrophages (MHC II^+^), the remaining Lin^−^ CD45^+^ cells were gated for CD3ϵ^low^ TCRβ^+^, CD3ϵ^int^ TCRβ^−^, CD3ϵ^high^ TCRβ^-^ which correspond to αβ T cells, γδ T cells, and DETC. **(B–D)** Comparison of the frequencies of three groups of T cells in WBC in the lesioned skin of WT and Zdhhc2^−/−^ mice after 4 and 8 days imiquimod stimulation. **(E–G)** Comparison the absolute cell number of αβ T cells, γδ T cells, and DETC in the psoriatic skin of WT and Zdhhc2^−/−^ mice after 4 and 8 days imiquimod stimulation. **(H–J)** MFI of CD44 for three groups of T cells in the lesioned skin of WT and Zdhhc2^−/−^ mice after 4 and 8 days imiquimod stimulation. Experiments were repeated twice, involving 3 mice for each time point per genotype (mean ± SEM). ^*^ *p* < 0.05, ^**^ *p* < 0.01, ^***^ *p* < 0.001, ^****^ *p* < 0.0001, unpaired Student’s *t-*test.

As T cells are the key effectors for self-reactivity in various autoimmune diseases including psoriasis, and the dendritic cells are generally required for the priming of T cell response. We reasoned that the blockade of pDC infiltration by Zdhhc2 deficiency in psoriatic skin could result in dampened T cell activity. Indeed a recent study showed that type I interferon producing pDCs were responsible for helper T cell activation (24). In addition, pDCs were found to drive γδ T cell activation through cytokine secretion such as IFN-α and TNF-α (25). Because we have demonstrated that pDC infiltration and IFN-α production was significantly inhibited in psoriatic skin of Zdhhc2 deficient mice, we next sought to determine whether T cell activation in psoriatic lesions of Zdhhc2^−/−^ mice was also inhibited. To this end, we used flow cytometry to quantitate the T cell subsets including αβ T cells, γδ T cells, and dendritic epidermal T cells (DETC), and to analyze the expression of T cell activation marker CD44 in the inflamed skin of WT and Zdhhc2 deficient mice (**Figure 4A** and histogram not shown). Interestingly, we found that the frequency and cell counts of infiltrating αβ T and δγ T cells in Zdhhc2 deficient mice were significantly increased (**Figures 4B, C, E, F** **)**. However, the DETC were significantly reduced in frequency and number in the inflamed skin of mutant mice (**Figures 4D, G**), and expression of the T cell activation marker CD44 was significantly decreased in all the three types of T cells (**Figures 4H–J**). To further determine whether the loss of Zdhhc2 will affect the functions of the three types of T cells, the transfer experiment was performed by intravenous injection of purified lymph node CD45.2^+^ T cells into CD45.1^+^ CD3ϵ^−/−^ mice. However, previously studies showed that γδ T cells represent only 1~2% of all T cells in lymph nodes (26), and DETC are only restricted to the murine epidermis (27). Therefore, we mainly detected the absolute number of CD45.2^+^ total T cells and CD45.2^+^ αβ T cells in the inflamed skin (**Supplementary Figure 3A**). As shown in **Supplementary Figure 3B**, T cell transfer from Zdhhc2 deficient donors as CD45.2^+^ T cells did not display phenotypic differences from WT donor T cells in the CD45.1^+^ CD3ϵ^−/−^ recipient mice after 7 days of imiquimod treatment. Furthermore, the expression of T cell activation marker CD44 also exhibited no significantly changes in Zdhhc2 deficient CD45.2^+^ donors T cells compared to WT controls (**Supplementary Figure 3C**). Our results suggest that the deficiency of Zdhhc2 imposes inhibition of T cells in lesioned skin *via* pDCs.

### Plasmacytoid Dendritic Cells Intrinsically Requires zDHHC2 for Cytokine Production

**Figure 5:**
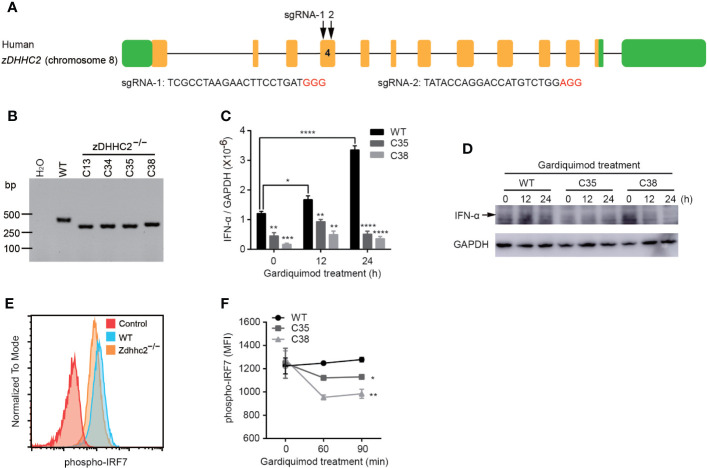
Inhibition of IFN-α production through IRF7 phosphorylation in CAL-1 zDHHC2^−/−^ cells. **(A)** Schematic representation of sgRNA-1 and sgRNA-2 sequences used to target exon 4 of the *zDHHC2* gene. Protospacer adjacent motif (PAM) sequences are shown in red color. **(B)** *zDHHC2* deficiency clone C13, C34, C35, and C38 were selected by PCR and agarose electrophoresis. Results from CAL-1 zDHHC2^−/−^ cells are shown and compared to CAL-1 WT cells. H_2_O was used as negative control. **(C, D)** qRT-PCR and western blot analysis of IFN-α expression in CAL-1 WT and CAL-1 zDHHC2^−/−^ cells with or without gardiquimod stimulation. **(E, F)** Flow cytometry analysis of phospho-IRF7 in CAL-1 WT and zDHHC2^−/−^ cells after gardiquimod stimulation. **(E)** Representative histogram of IRF7 phosphorylation level at 60 min after gardiquimod stimulation. **(F)** MFI of phospho-IRF7 at 0, 60, and 90 min after gardiquimod treatment, compared between WT and zDHHC2^−/−^ cells (n = 3, mean ± SEM). ^*^ *p* < 0.05, ^**^ *p* < 0.01, ***p < 0.001, ****p < 0.0001, unpaired Student’s *t-*test.

In our study, we found profound decrease of pDCs in psoriatic skin in Zdhhc2 deficient mice, and we further aimed to elucidate the cell type specific impact of zDHHC2 on pDCs function. Therefore, we used a human pDC cellular model, CAL-1 cells and performed CRISPR/Cas9 mediated genetic deletion of *zDHHC2* in this cell line (28). Interestingly, previous studies have shown that IFN-α production and activation of pDCs depend on phosphorylation of IRF7 (29). The specific knockout of IRF7 in mouse pDCs caused it almost lose the ability to produce IFN-α (30, 31). Additionally, the deficiency of IRF7 in human also significantly inhibit the production of IFN-α, making human susceptible to influenza virus infection (32). Therefore it was tempting to analyze the consequence of zDHHC2 deficient human pDCs by assessing cytokine production and IRF7 phosphorylation. First, we managed to obtain the zDHHC2 deficient CAL-1 cells following protocols in our previous studies (16, 18), using transient expression of CRISPR/Cas9 with fluorescent reporters and two sgRNAs (**Figure 5A**). PCR screening and sanger sequencing results showed that we have successfully established the CAL-1 zDHHC2^−/−^ cell line (**Figure 5B** and **Supplementary Figure 4A**). Then the mRNA level of IFN-α was detected in CAL-1 WT and CAL-1 zDHHC2^−/−^ cells after gardiquimod stimulation for 0, 12, and 24 h, respectively. Although CAL-1 cells have been reported to produce a small amount of IFN-α (28), we still observed that the mRNA expression level of IFN-α in WT cells exhibited a dramatic increase after gardiquimod stimulation while zDHHC2^−/−^ cells did not change significantly (**Figure 5C**). In addition, by exposing the PVDF membrane of the western blot experiment for up to 5 min, we observed that the protein expression level of IFN-α also increased in WT cells whereas zDHHC2^−/−^ cells had no changes after gardiquimod stimulation (**Figure 5D**). On the other hand, we compared the protein level of TLR7, and the phosphorylation level of IRF7 and p65 in CAL-1 WT and CAL-1 zDHHC2^−/−^ cells. Interestingly, the expression level of TLR7 and the phosphorylation level of p65 had no change in zDHHC2^−/−^ cells when compared to WT controls with or without gardiquimod stimulation (**Supplementary Figures 4B, C, E, F**). Moreover, zDHHC2 knockout did not affect intracellular distribution of TLR7 in CAL-1 cells with or without gardiquimod stimulation (**Supplementary Figure 4D**). However, compared with CAL-1 WT cells, the phosphorylation level of IRF7 exhibited significantly decreased in mutant cells after gardiquimod stimulation (**Figures 5E, F**). The results above provided evidence that zDHHC2 deficient CAL-1 cells had inhibited IFN-α production through reduced IRF7 phosphorylation.

## Discussion

Palmitoylation is a reversible lipid post-translational modification, which is regulated by palmitoyltransferases (also known as zDHHC family genes) and depalmitoylating enzymes, through adding a 16-carbon palmitoyl group onto cysteine ​​residues of proteins *via* a transient thioester bond (33). Published studies have shown that zDHHC family genes are closely related to the occurrence of diseases, such as neurodegenerative diseases and inflammatory disorders (6–9), and psoriasis is one of the chronic inflammatory skin diseases mediated by the immune cells and molecules of the innate and adaptive immune systems (10). However, prior to the current study, the role of zDHHC family genes in psoriasis has not been reported. In the present study, we found that the mRNA level of *Zdhhc2* was significantly elevated in mice inflamed skin upon imiquimod-induced psoriasis which suggested that there is a potential correlation between *Zdhhc2* gene and the occurrence of psoriasis. On this basis, we performed genetic deletion of *Zdhhc2* in C57BL/6 mice by CRISPR/Cas9 system, and first delineated that the knockout of *Zdhhc2* in mice was sufficient to mitigate psoriasis. To further understand the effect of Zdhhc2-deficiency on psoriasis, we performed H&E staining and flow cytometry analysis of psoriatic skin from WT and Zdhhc2^−/−^ mice. Strikingly, we found that deletion of *Zdhhc2* gene resulted in an extreme inhibition of inflammation response in mice ear skin which caused the inhibition of WBC infiltration in the psoriatic lesions of mice. Moreover, qRT-PCR results showed that Zdhhc2 deficiency significantly reduced the expression level of pro-inflammatory cytokines especially IFN-α in the inflamed skin. Yet, these results demonstrated that Zdhhc2 plays an important role in inflammatory response.

Evidences suggest that pDC can be detected in the spleen, lymph nodes, and blood but is almost undetectable in normal skin, however, when it infiltrates into the skin, it plays a crucial role in the initial stage of psoriasis by secreting large amounts of IFN-α (5, 20–23). In the present study, we observed that Zdhhc2 deficiency did not affect the frequency, absolute number, and activation state of pDC in the skin, spleen, DLN, and blood under steady-state conditions, showed that knockout of Zddhc2 does not affect the function of pDC under normal conditions. However, the frequency and absolute number of pDC were drastically increased in the lesioned skin, spleen, and DLN in WT mice after 8 days imiquimod treatment. Interestingly, Zdhhc2 deficiency mice exhibited significantly lower frequency and count of pDC than the WT mice, indicating that loss of Zdhhc2 potently inhibits the infiltration of pDC to organs, especially the skin, thereby reducing the incidence of psoriasis. Furthermore, using the CAL-1 cell line, we confirmed that zDHHC2 deficiency exhibited inhibition of IFN-α production, indicating the key role of zDHHC2 in inflamed skin infiltration and type-I interferon production of pDCs. It has been reported that palmitoylation was regulated by zDHHC family genes to control substrate protein-membrane association and have cross-talk with phosphorylation (34, 35). Interestingly, our study showed that the protein expression level of TLR7 which located on the endosomal membrane did not change in the absence of zDHHC2, and the intracellular distribution of TLR7 was also not affected. p65 activation is a critical signaling event in pDC mediated inflammatory response, however, we found that the phosphorylation level of p65 did not exhibit significant change in zDHHC2 deficient CAL-1 cells. Instead we found that loss of zDHHC2 in human pDCs significantly reduced IRF7 phosphorylation, which is a crucial transcription factor regulating IFN-α production (31, 32). In previous study, the CAL-1 human pDCs was found not robust in IFN-α secretion (28). In our study, although IFN-α shows extremely low expression level, following gardiquimod treatment, the WT cell exhibited significantly increase in IFN-α mRNA and protein expression, but strikingly such increases was completely abolished in zDHHC2 knockout CAL-1 cells. Our results showed that zDHHC2 is required by pDCs to produce IFN-α involving IRF7 phosphorylation, however we did not find total expression, intracellular distribution, or phosphorylation changes in more molecules such as TLR7 and p65. Therefore further studies are still necessary to elucidate how zDHHC2 deficiency could dampen IRF7 activation.

It has been reported that T cells also play an important role in the occurrence of psoriasis, and IFN-α produced by pDCs induces T cell activation (5, 24, 25). Our results showed that deletion of Zdhhc2 in mice dramatically decrease the activation level of all three subsets of T cells including αβ T cells, γδ T cells, and DETC in psoriatic skin. Our new findings in Zdhhc2 deficient mice that both IFN-α production and T cell activation were significantly inhibited, were consistent with results from previous studies showing that IFN-α produced by pDCs was critical to T cell activation (24, 25). Other reports indicate that in vitiligo and systemic lupus erythematosus, IFN-α-producing pDCs were also exhibited accumulation in perilesional skin, leading to T cells activation and recruitment (36–38). And in alopecia areata and pityriasis lichenoides, pDCs enhanced Th1-biased cellular immune responses, such as cytotoxic CD8^+^ T-cell function (39–41). These results indicate that the production of IFN-α by pDC to promote the enrichment and activation of T cells is similar in different inflammatory skin disorders.

In conclusion, our study identified the critical role of Zdhhc2 in development of psoriasis and pDC accumulation in the skin and we found that IFN-α production was completely dependent on Zdhhc2. The functional identification of zDHHC2 and mechanistic discoveries in our study suggest that manipulating the enzymatic activity of zDHHC2 might be a potential option for treatment of psoriasis and pDCs mediated inflammatory diseases.

## Data Availability Statement

The original contributions presented in the study are included in the article/**Supplementary Material**. Further inquiries can be directed to the corresponding author.

## Ethics Statement

The animal study was reviewed and approved by committee on animal care at Xinxiang Medical University.

## Author Contributions

BZ and EK wrote the manuscript. LL and ST established the Zdhhc2^−/−^ mice. LZ, QL, and ZL established the CAL-1-zDHHC2^−/−^ cell line. BZ, WY, WL, LH, RH, YW, and YG performed the experiments. TC and TJ managed the breeding of Zdhhc2^−/−^ mice. PP and MT provided the CAL-1 cell line. YL and EK extensively revised and edited the manuscript. All authors contributed to the article and approved the submitted version.

## Funding

This work was supported by the National Natural Science Foundation of China (Grant No. 31770824 and 32000491).

## Conflict of Interest

The authors declare that the research was conducted in the absence of any commercial or financial relationships that could be construed as a potential conflict of interest.

## Supplementary Material

Supplementary Figure 1 Genetic deletion of *Zdhhc2* gene in C57BL/6 mice. **(A)** Schematic diagram of the *Zdhhc2*-targeting sgRNA sequences, which were placed in *Zdhhc2* exon 3 and intron 3, respectively. Two PAMs sequences are shown in red color. **(B)** DNA sequencing analysis showed the presence of the intended *Zdhhc2* knockout mutation in F0 mice. The deletion size is indicated below the WT sequence. Red letters correspond to the PAM sequences and blue letters to the sgRNA sequences. The sequence of exon 3 was marked in gray.

Supplementary Figure 2 Gating strategy of 4 types of organs of pDC. **(A)** pDC gating strategy in the skin. After excluding dead cells (DAPI positive), T cells (CD5^+^), B cells (CD19^+^), and macrophages (F4/80^+^), the remaining cells were gated for CD11b^-^ Ly6C^+^ CD317^+^ as pDCs. **(B)** pDC gating strategy in the spleen. After excluding dead cells (DAPI positive), T cells (CD5^+^), B cells (CD19^+^), and macrophages (F4/80^+^), the remaining cells were gated for CD11b^-^ Siglec H^+^ CD317^+^ as pDCs. **(C)** pDC gating strategy in the DLN. After excluding dead cells (DAPI positive), T cells (CD5^+^), B cells (CD19^+^), and macrophages (CD169^+^), the remaining cells were gated for CD11b^-^ Siglec H^+^ CD317^+^ as pDCs. **(D)** pDC gating strategy in the blood. After excluding dead cells (DAPI positive), T cells (CD5^+^), B cells (CD19^+^), and monocytes (CD115^+^), the remaining cells were gated for CD11b^-^ Siglec H^+^ CD317^+^ as pDCs. The expression of pDC activation marker CD80 was displayed by histogram.

Supplementary Figure 3 Detection the effect of zDHHC2 knockout on T cell infiltration in inflamed skin by using transfer experiment. **(A)** CD45.2 T cells gating strategy in the skin. After excluding dead cells (DAPI positive), B cells (CD19^+^), neutrophil (Ly6G+), dendritic cells, and macrophages (MHC II^+^), the remaining cells were gated for CD45^+^ CD5^+^ as T cells or CD45^+^ TCRβ^+^ as αβ T cells, then T cells and αβ T cells were gated for CD45.1^−^ CD45.2^+^ as CD45.2 T cells and CD45.2 αβ T cells. **(B, C)** Comparison the absolute cell number and CD44 MFI of CD45.2^+^ T cells and CD45.2^+^ αβ T cells in the psoriatic skin of CD45.1^+^ CD3ϵ^−/−^ which respectively transferred with CD45.2^+^ T cells from WT and Zdhhc2^−/−^ mice. Experiments were repeated twice, involving three mice for each time point per genotype (mean ± SEM). ns, not significant.

Supplementary Figure 4 Detection of TLR7 protein level, intracellular distribution, and p65 phosphorylation level in WT and zDHHC2^−/−^ CAL-1 cells. **(A)** DNA sequencing analysis showed the presence of the intended zDHHC2^−/−^ cell lines. The deletion size is indicated below the WT sequence. Red letters correspond to the PAM sequences and blue letters to the sgRNA sequences. **(B)** MFI of TLR7 expression in CAL-1 WT and CAL-1 zDHHC2^−/−^ cells after stimulating by gardiquimod for indicated time points (n = 3, mean ± SEM). **(C)** Western blot analysis for TLR7 in CAL-1 WT and CAL-1 zDHHC2^−/−^ cells after stimulating by gardiquimod. **(D)** Immunofluorescence of CAL-1 WT and CAL-1 zDHHC2^−/−^ cells after 0 or 24 h gardiquimod stimulation. Scale bar = 5 μm. **(E)** MFI of p65 phosphorylation in zCAL-1 WT and CAL-1 zDHHC2^−/−^ cells after stimulating by gardiquimod for indicated time points (n = 3, mean ± SEM). **(F)** Western blot analysis for phospho-p65 in CAL-1 WT and CAL-1 zDHHC2^−/−^ cells after stimulating by gardiquimod. ns, not significant.

## Abbreviations

pDC, plasmacytoid dendritic cell; sgRNA, small guide RNA; WBC, white blood cells; DETC, dendritic epidermal T cells; H&E staining, hematoxylin and eosin staining; IFN-α, interferon-α; TNF-α, tumor necrosis factor-α; IL-23, interleukin-23; IL-17a, interleukin-17a; HPRT: hypoxanthine-guanine phosphoribosyltransferase; WT, wildtype; MFI, mean fluorescence intensity; IRF7, interferon regulatory factor 7; TLR7, toll-like receptor 7; PAM, protospacer adjacent motif; DLN, draining lymph nodes.
