# Supplementary figures and images for: Zdhhc2 Is Essential for Plasmacytoid Dendritic Cells Mediated Inflammatory Response in Psoriasis
Source: Front Immunol. 2021 Jan 8;11:607442. doi: 10.3389/fimmu.2020.607442 (PMC7819861; doi:10.3389/fimmu.2020.607442)

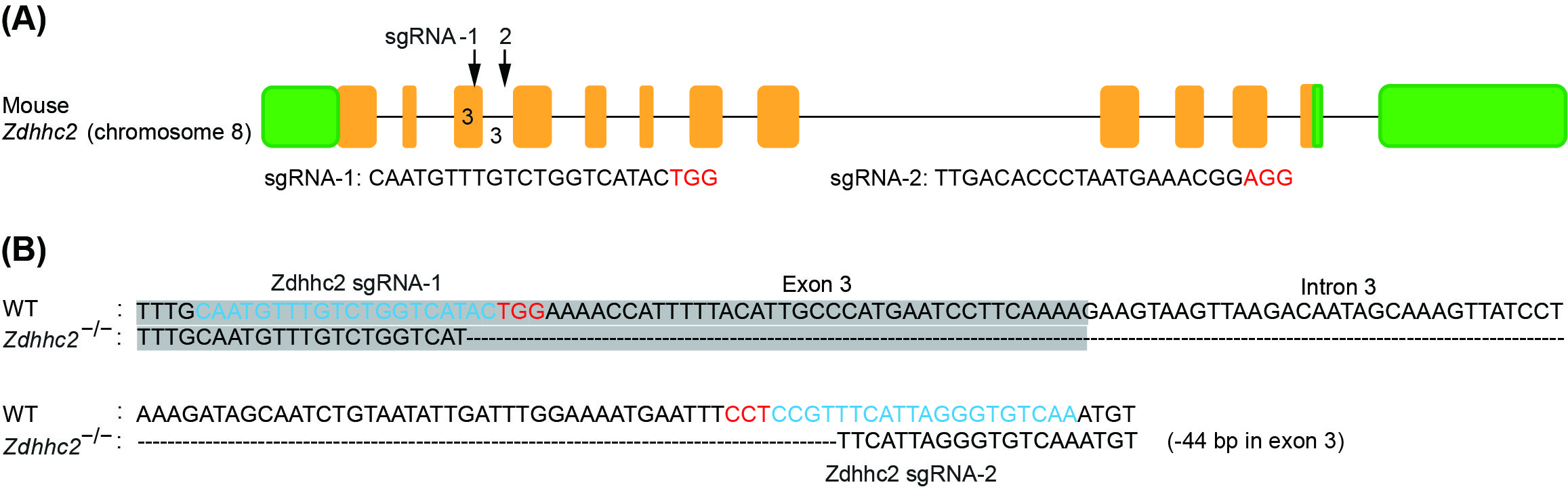

Supplement: Supplementary Figure 1 — Genetic deletion of Zdhhc2 gene in C57BL/6 mice. (A) Schematic diagram of the Zdhhc2-targeting sgRNA sequences, which were placed in Zdhhc2 exon 3 and intron 3, respectively. Two PAMs sequences are shown in red color. (B) DNA sequencing analysis showed the presence of the intended Zdhhc2 knockout mutation in F0 mice. The deletion size is indicated below the WT sequence. Red letters correspond to the PAM sequences and blue letters to the sgRNA sequences. The sequence of exon 3 was marked in gray. [file Image_1.jpeg]

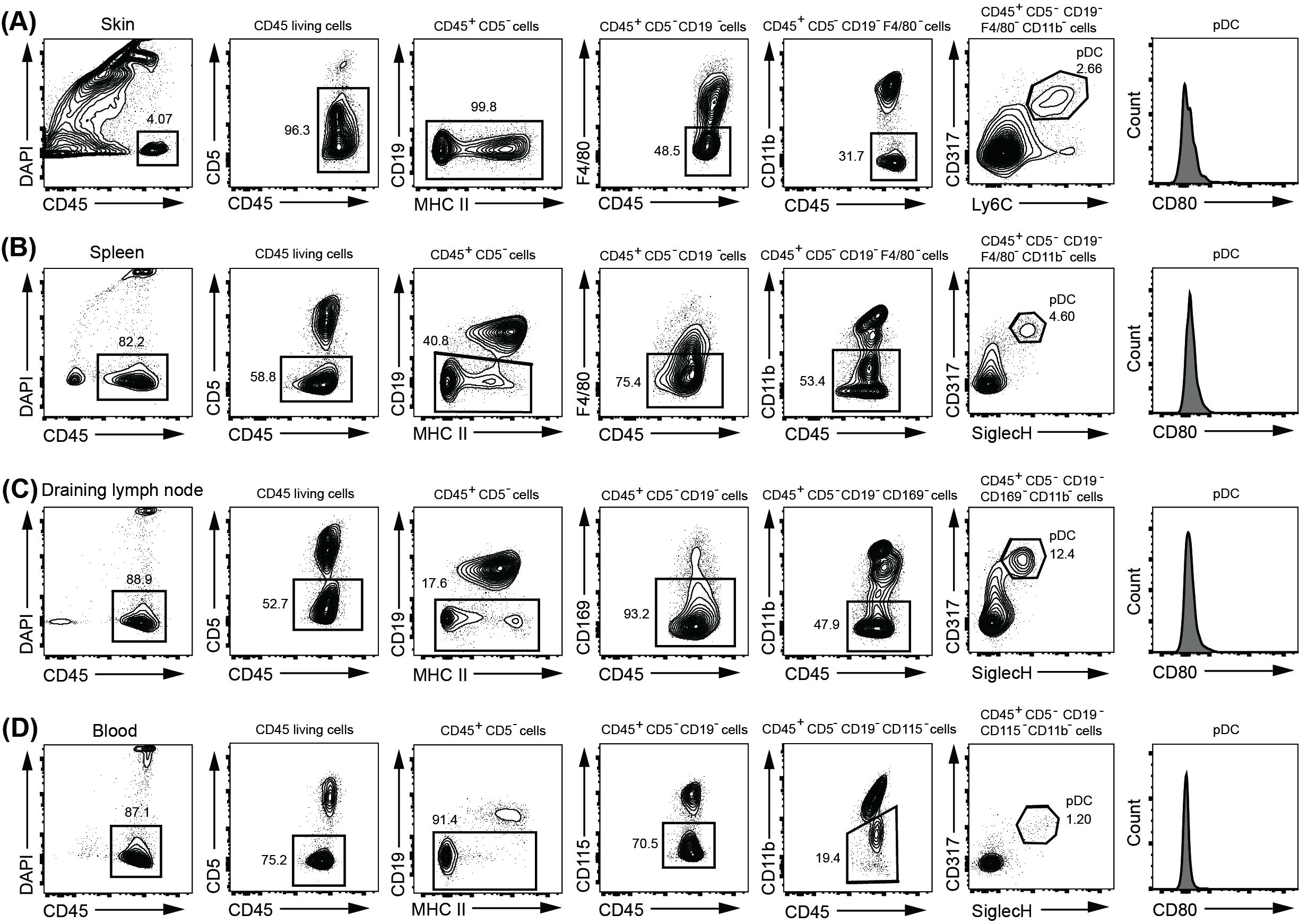

Supplement: Supplementary Figure 2 — Gating strategy of 4 types of organs of pDC. (A) pDC gating strategy in the skin. After excluding dead cells (DAPI positive), T cells (CD5+), B cells (CD19+), and macrophages (F4/80+), the remaining cells were gated for CD11b- Ly6C+ CD317+ as pDCs. (B) pDC gating strategy in the spleen. After excluding dead cells (DAPI positive), T cells (CD5+), B cells (CD19+), and macrophages (F4/80+), the remaining cells were gated for CD11b- Siglec H+ CD317+ as pDCs. (C) pDC gating strategy in the DLN. After excluding dead cells (DAPI positive), T cells (CD5+), B cells (CD19+), and macrophages (CD169+), the remaining cells were gated for CD11b- Siglec H+ CD317+ as pDCs. (D) pDC gating strategy in the blood. After excluding dead cells (DAPI positive), T cells (CD5+), B cells (CD19+), and monocytes (CD115+), the remaining cells were gated for CD11b- Siglec H+ CD317+ as pDCs. The expression of pDC activation marker CD80 was displayed by histogram. [file Image_2.jpeg]

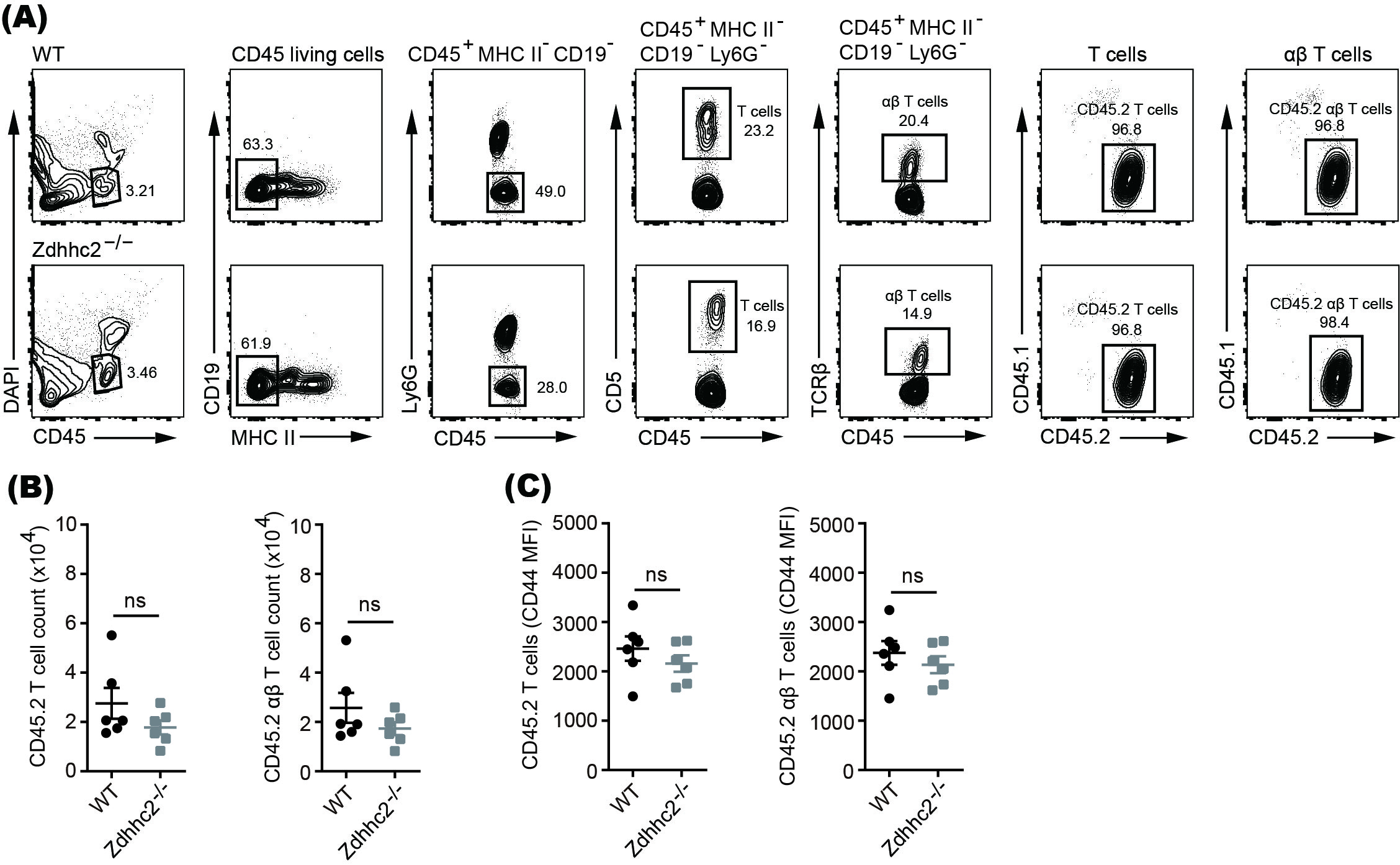

Supplement: Supplementary Figure 3 — Detection the effect of zDHHC2 knockout on T cell infiltration in inflamed skin by using transfer experiment. (A) CD45.2 T cells gating strategy in the skin. After excluding dead cells (DAPI positive), B cells (CD19+), neutrophil (Ly6G+), dendritic cells, and macrophages (MHC II+), the remaining cells were gated for CD45+ CD5+ as T cells or CD45+ TCRβ+ as αβ T cells, then T cells and αβ T cells were gated for CD45.1− CD45.2+ as CD45.2 T cells and CD45.2 αβ T cells. (B, C) Comparison the absolute cell number and CD44 MFI of CD45.2+ T cells and CD45.2+ αβ T cells in the psoriatic skin of CD45.1+ CD3ϵ−/− which respectively transferred with CD45.2+ T cells from WT and Zdhhc2−/− mice. Experiments were repeated twice, involving three mice for each time point per genotype (mean ± SEM). ns, not significant. [file Image_3.jpeg]

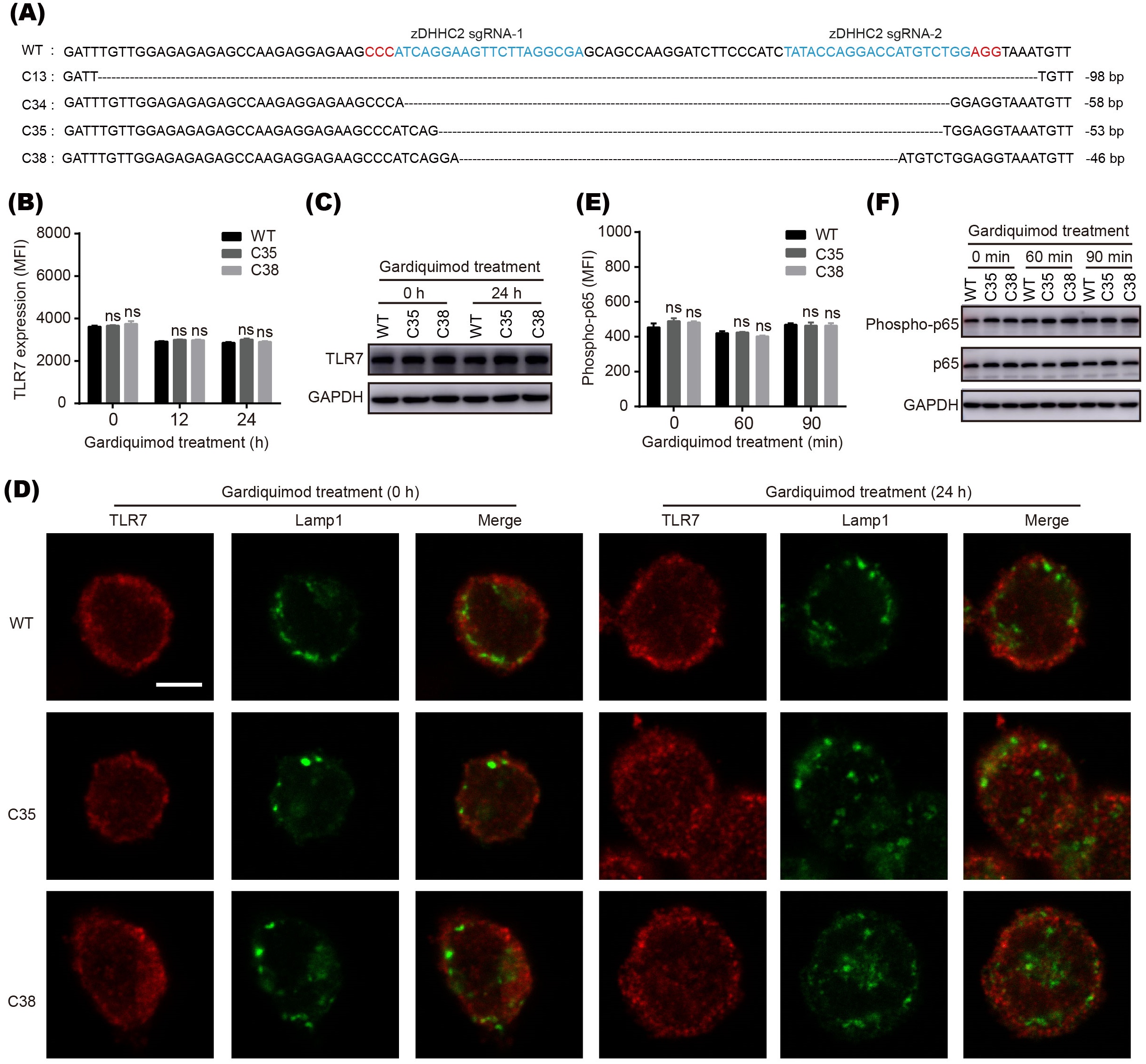

Supplement: Supplementary Figure 4 — Detection of TLR7 protein level, intracellular distribution, and p65 phosphorylation level in WT and zDHHC2−/− CAL-1 cells. (A) DNA sequencing analysis showed the presence of the intended zDHHC2−/− cell lines. The deletion size is indicated below the WT sequence. Red letters correspond to the PAM sequences and blue letters to the sgRNA sequences. (B) MFI of TLR7 expression in CAL-1 WT and CAL-1 zDHHC2−/− cells after stimulating by gardiquimod for indicated time points (n = 3, mean ± SEM). (C) Western blot analysis for TLR7 in CAL-1 WT and CAL-1 zDHHC2−/− cells after stimulating by gardiquimod. (D) Immunofluorescence of CAL-1 WT and CAL-1 zDHHC2−/− cells after 0 or 24 h gardiquimod stimulation. Scale bar = 5 μm. (E) MFI of p65 phosphorylation in zCAL-1 WT and CAL-1 zDHHC2−/− cells after stimulating by gardiquimod for indicated time points (n = 3, mean ± SEM). (F) Western blot analysis for phospho-p65 in CAL-1 WT and CAL-1 zDHHC2−/− cells after stimulating by gardiquimod. ns, not significant. [file Image_4.jpeg]
