# Supplementary material for: Zdhhc2 Is Essential for Plasmacytoid Dendritic Cells Mediated Inflammatory Response in Psoriasis
Source: Front Immunol. 2021 Jan 8;11:607442. doi: 10.3389/fimmu.2020.607442 (PMC7819861; doi:10.3389/fimmu.2020.607442)
Supplement: Supplementary file 5 [file Table_1.docx]

**Supplementary** **Table 1 | Antibodies for flow cytometry**

| **Species** | **Antigen** | **Fluorochrome** | **Catlog. No.** | **Supplier** |
| --- | --- | --- | --- | --- |
| Mouse | Anti-CD45 | BUV805 | 748370 | BD Biosciences |
|  | Anti-CD317 | APC | 127016 | Biolegend |
|  | Anti-Ly6C | BV785 | 128041 | Biolegend |
|  | Anti-F4/80 | PE-CY7 | 25-4801-82 | eBioscience |
|  | Anti-CD11b | BUV395 | 563553 | BD Biosciences |
|  | Anti-CD19 | BV750 | 115561 | Biolegend |
|  | Anti-CD5 | PE | 12-0051-83 | eBioscience |
|  | Anti-MHC II | APCeFluor780 | 47-5321-82 | eBioscience |
|  | Anti-CD80 | PE-Cy5 | 15-0801-82 | Invitrogen |
|  | Anti-Siglec H | FITC | 11-0333-82 | eBioscience |
|  | Anti-CD115  CD115 | PE-Cy7 | 25-1152-82 | eBioscience |
|  | Anti-CD169 | PE | 12-5755-82 | eBioscience |
|  | Anti-CD45 | APC-eFluor780 | 4331936 | eBioscience |
|  | Anti-MHCII | Alexa Fluor700 | 56-5321-82 | eBioscience |
|  | Anti-CD19 | Alexa Fluor700 | 56-0193-82 | eBioscience |
|  | Anti-Ly6G | Alexa Fluor700 | 561236 | BD Biosciences |
|  | Anti-CD3ε | PE | MABF317 | Millipore |
|  | Anti-TCRβ | FITC | 11-5961-85 | eBioscience |
|  | Anti-CD44 | Super Bright 600 | 63-0441-82 | eBioscience |

The table continues on the next page.

| Mouse | Anti-CD45.2 | FITC | 11-0454-85 | eBioscience |
| --- | --- | --- | --- | --- |
|  | Anti-CD45 | BV510 | 563891 | BD Biosciences |
|  | Anti-MHC II | eFlour450 | 48-5321-82 | eBioscience |
|  | Anti-CD45.1 | PE-Cy7 | 25-0453-82 | eBioscience |
|  | Anti-CD5 | APC | 17-0051-82 | eBioscience |
|  | Anti-TCRβ | APC-eFluor780 | 47-5961-82 | eBioscience |
| Human | Anti-TLR7 | PE | MA5-16249 | Invitrogen |
|  | Anti-p65 (phospho-Ser536) |  | 3033 | Cell signaling technology |
|  | Anti-IRF7 (phospho-Ser477) |  | YP1366 | Immunoway |
|  | Goat-α-Rabbit | APC | A-10931 | Thermo Fisher  Scientific |

**Supplementary** **Table 2 | Primer pairs used for qRT-PCR analysis**

| **Species** | **Gene name** | **Sequence (5’ to 3’)** |
| --- | --- | --- |
| Mouse | Zdhhc2-F | ACGGATTCAGCTTGGGTTTCA |
|  | Zdhhc2-R | GGCAAGTTGGAAAGGAACAGC |
|  | Zdhhc2-KO-F | TTTACATTGCCCATGAATCCTT |
|  | Zdhhc2-KO-R | TGGTCCTGGTGTAGATGGGA |
|  | IFN-α-F | ACCAGCAGCTCAATGACCTG |
|  | IFN-α-R | CTTCTGCTCTGACCACCTCC |
|  | TNF-α-F | GGTGCCTATGTCTCAGCCTC |
|  | TNF-α-R | ACTGATGAGAGGGAGGCCAT |
|  | IL-23-F | TATCCAGTGTGAAGATGGTTGTG |
|  | IL-23-R | CACTAAGGGCTCAGTCAGAGTTG |
|  | IL-17a-F | GAAGGCCCTCAGACTACCTCAA |
|  | IL-17a-R | CAGCTTTCCCTCCGCATTGAC |
|  | HPRT-F | CGTCGTGATTAGCGATGATG |
|  | HPRT-R | ACAGAGGGCCACAATGTGAT |
| Human | IFN-α-F | GACTCCATCTTGGCTGTGA |
|  | IFN-α-R | TGATTTCTGCTCTGACAACCT |
|  | GAPDH-F | GCATCCTGGGCTACACTGAG |
|  | GAPDH-R | CCACCACCCTGTTGCTGTAG |
